# Characterization of myocardial T_1_ and partition coefficient as a function of time after gadolinium delivery in healthy subjects

**DOI:** 10.1186/1532-429X-13-S1-P31

**Published:** 2011-02-02

**Authors:** Kelvin Chow, Jacqueline A Flewitt, Jordin D Green, Matthias G Friedrich, Richard B Thompson

**Affiliations:** 1 University of Alberta, Edmonton, AB, Canada; 2 University of Calgary, Calgary, AB, Canada; 3 Siemens Healthcare, Calgary, AB, Canada

## Background

Diffuse myocardial fibrosis is associated with myocardial infarction [1], heart failure [2] and dilated cardiomyopathy [3]. Conventional T_1_-weighted late gadolinium enhancement (LGE) imaging highlights focal scaring in contrast to remote reference tissue, but it cannot detect global changes in T_1_ associated with diffuse fibrosis. Quantitative T_1_ imaging permits assessment of diffuse fibrosis by eliminating the use of reference tissue, but the dependence of the derived partition coefficient (lambda) on the time post-contrast injection (t_post_) is not well established.

## Purpose

Determine blood and myocardial T_1_ values as a function of t_post_ and the resulting dependence of the blood-tissue partition coefficient.

## Methods

Nine healthy subjects (22.0±5.5 yrs, 6 male) were imaged using a Siemens Avanto 1.5T MRI. T_1_ mapping was performed on a mid-ventricular short-axis slice using a custom saturation recovery single-shot TrueFISP sequence at baseline and one-minute intervals for 15 minutes following a bolus injection of gadopentetate dimeglumine (0.1 mmol/kg). At each time point, one "no-saturation" image and nine images with varying saturation recovery times spanning the cardiac cycle were acquired during a single breath-hold.

The myocardium was divided into 18 segments and mean values were fitted to a 3 parameter saturation recovery curve to determine T_1_ values for each segment at every time point. Blood T_1_ values were computed using a region of interest within the left ventricular cavity. Lambda was computed using {lambda=[R_1_(myocardium_post_) - R_1_(myocardium_pre_)]/[R_1_(blood_post_) - R_1_(blood_pre_)]}, where R_1_=1/T_1_.

## Results

**Figure 1:**
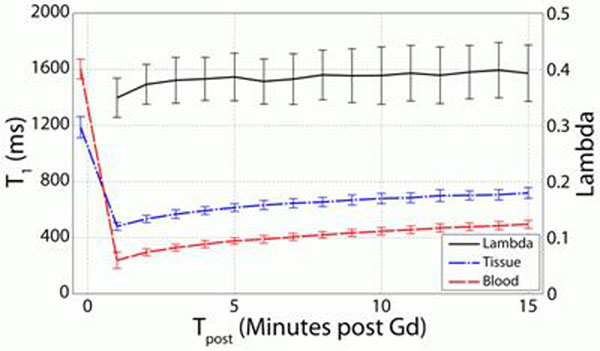
T_1_ (myocardium, blood) and lamba following contrast injection.

Figure 1 shows myocardial T_1_, blood T_1_, and lambda values averaged over all segments and subjects as a function of t_post_. Average within-subject standard deviations of T_1_ and lambda for t_post_ from 3-15 min were 34.1 ms and 0.046 respectively. Linear regression for lambda and t_post_ (3-15 min) shows an increase in lambda of 0.001 min^-1^ (R^2^=0.75). Quantitative T_1_ imaging is likely to be added to a clinical protocol following LGE imaging (t_post_ 10-15 min), where T_1_ values increase by 5.9±1.6% and lambda increase by 1.1±2.7%.

## Conclusion

Saturation recovery SSFP T_1_ mapping can be performed in a single breath-hold with derived blood-tissue partition coefficient (lambda) values in good agreement with previous measurements^3^. In the post-LGE window of 10-15 min after contrast bolus, derived lambda values show less time dependence than myocardial T_1_.

## References

[B1] Flacke SJ Radiol 2001 10.1148/radiology.218.3.r01fe18703 11230643

[B2] Iles L J Am Coll Cardiol 2008 19007595 10.1016/j.jacc.2008.06.049

[B3] Jerosch-Herold M Am J Physiol Heart Circ Physiol 200818660445

